# Determinants of excess mortality during the COVID-19 pandemic in 18 countries of the CMOR consortium

**DOI:** 10.1093/eurpub/ckac129.278

**Published:** 2022-10-25

**Authors:** C Kneebone-Hopkins, A Artemiou, CA Demetriou

**Affiliations:** 1 School of Mathematics, University of Cardiff, Cardiff, UK; 2 Department of Primary Care and Population Health, University of Nicosia Medical School, Nicosia, Cyprus

## Abstract

Many countries suffered excess all-cause mortality during the COVID-19 pandemic. This study aims to identify factors associated with excess mortality rates (EMR) in partaking countries during 2020. Weekly all-cause death counts for 2015-2020 were extracted from national databases for Australia, Austria, Brazil, Cyprus, Denmark, Estonia, France, Georgia, Israel, Italy, Mauritius, Norway, Peru, Slovenia, Sweden, USA, Ukraine and UK. EMR per 100,000 population were gauged using a 5-year mean baseline. Separate OLS multiple linear regressions explored pre-pandemic country profiles including healthcare system, geographic, socio-economic and population factors. Feature selection methods detected the main factors contributing to 2020 EMR. The health system model showed that an extra nurse per 1,000 and a 1% increase in Healthcare Access and Quality Index reduces EMR by 41.7% (p = 0.019) and 0.48% (p = 0.034). The model was statistically significant (R^2=0.415,p=0.018). Although the geographical model suggested that a 1% increase in neighbouring countries increased EMR by 0.42% (p = 0.078), population density and the model itself were statistically insignificant (p > 0.05). The socio-economic and population model indicated a 1% increase in service employed (% of employed) and investment (% GDP) was linked with a 43.4% (p = 0.01) and 43.7% (p = 0.01) fall in EMR. The model was significant (R^2=0.488, p = 0.007). Death registration quality and population share over 70 years, improved model performance (R^2=0.632), but neither approached nominal significance. EMR during the COVID-19 pandemic benefited from higher ratios of nurses to population and able and prompt healthcare. The geographic traits were trivial in explaining EMR variation. Higher ratios of service employed, and investment (% of GDP) were linked to lower EMR. These results help to inform policies now and in future pandemics to strengthen resilience against EMR.

**Key messages:**

• This study identified which pre-pandemic factors affected EMR in partaking countries, adding to a growing body of work on the COVID-19 pandemic.

• Higher ratios of nurses to population, able and prompt healthcare, higher % employed, and investment (% of GDP) were linked to lower EMR.

